# Appearance of COVID-19 pneumonia on 1.5 T TrueFISP MRI

**DOI:** 10.1590/0100-3984.2021.0028

**Published:** 2021

**Authors:** Judith Eva Spiro, Adrian Curta, Shiwa Mansournia, Constantin Arndt Marschner, Stefan Maurus, Ludwig Thomas Weckbach, Dennis Martin Hedderich, Julien Dinkel

**Affiliations:** 1 Department of Radiology, University Hospital, LMU Munich, Munich, Germany.; 2 Department of Medicine I, University Hospital, LMU Munich, Munich, Germany.; 3 Department of Neuroradiology, Technical University of Munich, School of Medicine, Munich, Germany.; 4 Comprehensive Pneumology Center (CPC-M), Member of the German Center for Lung Research (DZL), Munich, Germany.; 5 Department of Radiology, Asklepios Lung Center Munich-Gauting, Gauting, Germany.

**Keywords:** Thorax, Tomography, spiral computed, Magnetic resonance imaging, Pneumonia, Coronavirus infections, Tórax, Tomografia computadorizada, espiral, Ressonância magnética, Pneumonia, Infecções por coronavírus

## Abstract

**Objective:**

To evaluate the performance of 1.5 T true fast imaging with steady state precession (TrueFISP) magnetic resonance imaging (MRI) sequences for the detection and characterization of pulmonary abnormalities caused by coronavirus disease 2019 (COVID-19).

**Materials and Methods:**

In this retrospective single-center study, computed tomography (CT) and MRI scans of 20 patients with COVID-19 pneumonia were evaluated with regard to the distribution, opacity, and appearance of pulmonary lesions, as well as bronchial changes, pleural effusion, and thoracic lymphadenopathy. McNemar’s test was used in order to compare the COVID-19-associated alterations seen on CT with those seen on MRI.

**Results:**

Ground-glass opacities were better visualized on CT than on MRI (*p* = 0.031). We found no statistically significant differences between CT and MRI regarding the visualization/characterization of the following: consolidations; interlobular/intralobular septal thickening; the distribution or appearance of pulmonary abnormalities; bronchial pathologies; pleural effusion; and thoracic lymphadenopathy.

**Conclusion:**

Pulmonary abnormalities caused by COVID-19 pneumonia can be detected on TrueFISP MRI sequences and correspond to the patterns known from CT. Especially during the current pandemic, the portions of the lungs imaged on cardiac or abdominal MRI should be carefully evaluated to promote the identification and isolation of unexpected cases of COVID-19, thereby curbing further spread of the disease.

## Abstract (pt)

**Objetivo:**

Avaliar o desempenho da ressonância magnética (RM) de 1,5 T *true fast imaging with steady state precession* (TrueFISP) para detecção e caracterização de anormalidades pulmonares causadas por doença do coronavírus 2019 (COVID-19).

**Materiais e Métodos:**

Neste estudo retrospectivo unicêntrico, imagens de tomografia computadorizada (TC) e RM de 20 pacientes com pneumonia COVID-19 foram avaliadas em relação a distribuição, opacidade e forma das lesões pulmonares, anormalidades brônquicas, derrame pleural e linfadenopatia torácica. O teste de McNemar foi usado para comparar os achados associados à COVID-19 na TC e na RM.

**Resultados:**

As opacidades em vidro fosco foram mais bem visualizadas na TC do que na RM (*p* = 0,031). Não foram encontradas diferenças estatisticamente significantes entre TC e RM em relação aos outros aspectos, ou seja, visualização de consolidações e espessamento septal interlobular/intralobular, distribuição ou forma de anormalidades pulmonares, doenças brônquicas, derrame pleural ou linfadenopatia torácica.

**Conclusão:**

As anomalias pulmonares causadas pela pneumonia por COVID-19 podem ser detectadas nas sequências TrueFISP e correspondem aos padrões conhecidos da TC. Especialmente em face da pandemia atual, as porções de imagem dos pulmões na RM cardíaca ou abdominal devem ser cuidadosamente avaliadas para apoiar a identificação e o isolamento de casos inesperados de COVID-19 e, assim, conter a disseminação.

## INTRODUCTION

Coronavirus disease 2019 (COVID-19) was first described in December 2019 in the city of Wuhan, China. Since then, infection with severe acute respiratory syndrome coronavirus 2 (SARS-CoV-2) has been spreading rapidly around the globe, causing a pandemic that, by the end of January 2021, resulted in more than 100 million confirmed cases and over 2 million confirmed deaths^(1)^.

Approximately 18% of people infected with SARS CoV-2 are asymptomatic, the remainder developing symptoms such as fever, cough, and shortness of breath^(2)^. The last symptom in particular can be attributed to pneumonia caused by SARS-CoV-2, which in some patients has a severe course and may lead to death, especially in individuals with pre-existing medical conditions and in the elderly^(2,3)^. In addition, complications such as pulmonary artery embolism and myocardial injury worsen the prognosis of affected patients^(4,5)^.

The current gold standard for diagnosing COVID-19 is pathogen detection using reverse transcriptase polymerase chain reaction (RT-PCR) of nose/throat swab or sputum samples. However, computed tomography (CT) also plays an important role because of its high sensitivity for the detection of atypical pneumonia^(2,6)^. A large number of studies have described the morphology of COVID-19 pneumonia on chest CT images. In the early stage of the infection, it typically includes bilateral, multilobar ground-glass opacities, predominantly in the subpleural space and lung bases. In the later stages, consolidations and linear opacities can be observed, as can thickened interlobular and intralobular septa, as well as the mosaic (crazy-paving) pattern and the reverse halo sign^(7-9)^.

Because COVID-19 is highly contagious and is transmitted through droplets, early identification and immediate isolation of infected individuals are of great importance in containing the pandemic. This procedure is complicated by the fact that many patients who are asymptomatic or have nonspecific symptoms are unaware of their infection and unwittingly spread the virus^(2,10,11)^. It is therefore particularly important to identify and isolate those patients whenever possible. Radiologists play a key role because signs of COVID-19 pneumonia may be incidental findings on radiological examinations^(12,13)^.

Although the potential of magnetic resonance imaging (MRI) for pulmonary imaging has been highlighted in a variety of studies, the suitability of MRI for the detection of COVID-19 pneumonia in particular has only occasionally been investigated^(12-19)^. Given that standard cardiac MRI protocols usually include sequences that cover both lungs, the examination is well suited for the detection of asymptomatic patients with pulmonary manifestations of COVID-19. We therefore evaluated whether pulmonary abnormalities caused by COVID-19 pneumonia can be seen on cardiac MRI and whether MRI scans show the pattern typically seen on CT.

## MATERIALS AND METHODS

This was a retrospective single-center study. The study was approved by the research ethics committee of our institution. Due to the retrospective nature of the study, the requirement for written informed consent was waived.

### Study population

We included patients with RT-PCR-confirmed COVID-19 who had undergone chest CT and cardiac MRI between March 18 and May 5, 2020. In all patients, the indication for performing cardiac MRI was an elevated troponin T level on a high-sensitivity troponin T assay, assuming that coronary artery disease had been excluded. Significant coronary artery disease was ruled out by coronary CT angiography or cardiac catheter examination within the last 24 months. A total of 20 patients (18 males and 2 females) met the inclusion criteria. The mean age was 64.8 ± 13.9 years (range, 27-82 years). Two patients had a reduced left ventricular ejection fraction (27% and 29%, respectively), and another patient presented with sinus tachycardia (117 bpm). The other patients did not show signs of cardiac dysfunction. Clinical and imaging findings were obtained from patient medical records. In cases in which more than one CT scan of the chest was acquired, the scan chronologically closest to the date of the MRI examination was analyzed.

### Chest CT

Fifteen CT examinations with low-dose protocols were performed to evaluate pulmonary changes due to COVID- 19 pneumonia. Four contrast-enhanced CT scans were performed to rule out pulmonary embolism. One CT scan was performed for image-guided positioning of a chest tube due to large pleural effusion. Eighteen CT scans were performed on a Somatom Definition AS+ scanner (Siemens Healthineers, Erlangen, Germany), one was performed on a Somatom Force scanner (Siemens Healthineers) and one was performed on an Optima CT660 scanner (GE Healthcare, Milwaukee, WI, USA) scanner. The scan protocols were as follows: mean tube current of 93.4 ± 66.1 mAs (range, 37-232 mAs); mean tube voltage of 108.0 ± 12.0 kVp (range, 80-120 kVp); mean slice thickness of 1.7 ± 0.9 mm (range, 1-3 mm); mean volume CT dose index of 3.9 ± 1.3 mGy (range, 0.8-15.3 mGy); and mean dose-length product (mGy × cm) of 131.6 ± 102.0 (range, 26-495).

### Thoracic MRI

All MRI examinations were performed in the setting of suspected myocardial injury and in a Magnetom Aera 1.5 T scanner (Siemens Healthineers). The standard cardiac protocol at our department includes coronal, axial, and sagittal true fast imaging with steady state precession (TrueFISP) sequences covering the entire thorax, in order to evaluate the pulmonary changes. The scan parameters of the sequences were as follows: echo time, 1.38-1.65 ms; repetition time, 441.59 ms; flip angle, 80°; slice thickness, 6 mm; field of view, 340 × 276 mm; and matrix, 256 × 178.

### Image analysis

Two radiologists (with 7 and 14 years of experience, respectively) reviewed the chest CT and MRI scans on a picture archiving and communication system workstation (Syngo Studio version VB36E; Siemens Healthcare, Erlangen, Germany). Discrepancies were resolved by consensus. To avoid memory bias, the analyses of the CT and MRI scans were conducted with a two-week interval between them.

All scans were evaluated in axial, coronal, and sagittal orientation. For evaluation of CT images, lung window settings (width, 1600 HU; level, −600 HU) and soft-tissue window settings (width, 300 HU; level, 40 HU) were applied.

Pulmonary findings on CT images were described according to the Fleischner Society glossary of terms for thoracic imaging^(20)^. Lung abnormalities observed on MRI were described according to the current literature on the subject, which adopts the CT terminology outlined in the Fleischner Society glossary. For instance, we defined ground-glass opacities as a hazy increase in signal intensity in the lung, with preserved bronchial and vascular margins^(14,16-19,21,22)^. The two readers evaluated the distribution of the pulmonary findings (unilateral vs. bilateral; unilobar vs. multilobar; affected lobes; involvement of upper, middle, and lower zones; peripheral or central lung involvement; anterior or posterior lung involvement; and subpleural sparing), as well as their opacity (ground-glass opacities, consolidation, and interlobular/intralobular septal thickening) and appearance (patchy/segmental, rounded, crazy-paving, halo/reversed halo, linear/reticular, and air bronchogram). The readers also evaluated the bronchial changes (bronchiectasis, bronchial wall thickening, and mucus plugging), as well as looking for pleural effusion and thoracic lymphadenopathy. Regarding distribution, the inner two thirds and the outer third of the lung were defined as the central zone and the peripheral zone, respectively. A virtual horizontal line passing through the middle of the lung in the axial plane separated the anterior zone from the posterior zone, and two additional virtual horizontal lines dividing the lungs into equal thirds in the coronal plane delineated the upper, middle, and lower zones. The MRI findings were compared with the CT findings and with those in the current literature on COVID-19.

### Statistical analysis

Statistical analysis was performed with the SPSS Statistics software package, version 25.0 (IBM Corp., Armonk, NY, USA). Quantitative variables are expressed as mean ± standard deviation and range, and categorical variables are expressed as absolute and relative frequencies. McNemar’s test was used in order to compare COVID-19-associated findings on CT and MRI scans. The level of significance was set at α = 0.05.

## RESULTS

Twenty patients with RT-PCR-proven COVID-19, all of whom had undergone CT and MRI examination of the chest between March 18 and May 5, 2020 at our institution, met the inclusion criteria. Demographic data and clinical symptoms of the study population at first admission are presented in Table 1.

**Table 1:** Demographic data and clinical symptoms of the patients included, at first admission.

Variable	(N = 20)
Gender, n (%)	
Male	18 (90)
Female	2 (10)
Age (years), mean ± SD (range)	64.8 ± 13.9 (27-82)
Symptoms, n (%)	
Fever	14 (70)
Cough	12 (60)
Fatigue	10 (50)
Dyspnea	6 (30)
Nausea/emesis	4 (20)
Headache	3 (15)
Diarrhea	2 (10)
Abdominal pain	1 (5)
Chest pain	1 (5)

The mean interval between the first RT-PCR that was positive for SARS-CoV-2 and the CT examination was 8.1 ± 11.7 days (range, −3 days [CT performed prior to RT-PCR] to 40 days). The mean interval between the first positive RT-PCR and the MRI examination was 17.2 ± 11.7 days (range, 4-39 days). The mean interval between CT and MRI was 9.0 ± 9.0 days (range, −7 days [MRI performed prior to CT] to 27 days). Twelve patients underwent only one MRI examination, and eight underwent an additional (follow-up) MRI, at an average of 42.0 ± 7.4 days (range, 27-51 days) after the first.

### Imaging findings

**Figure 1:**
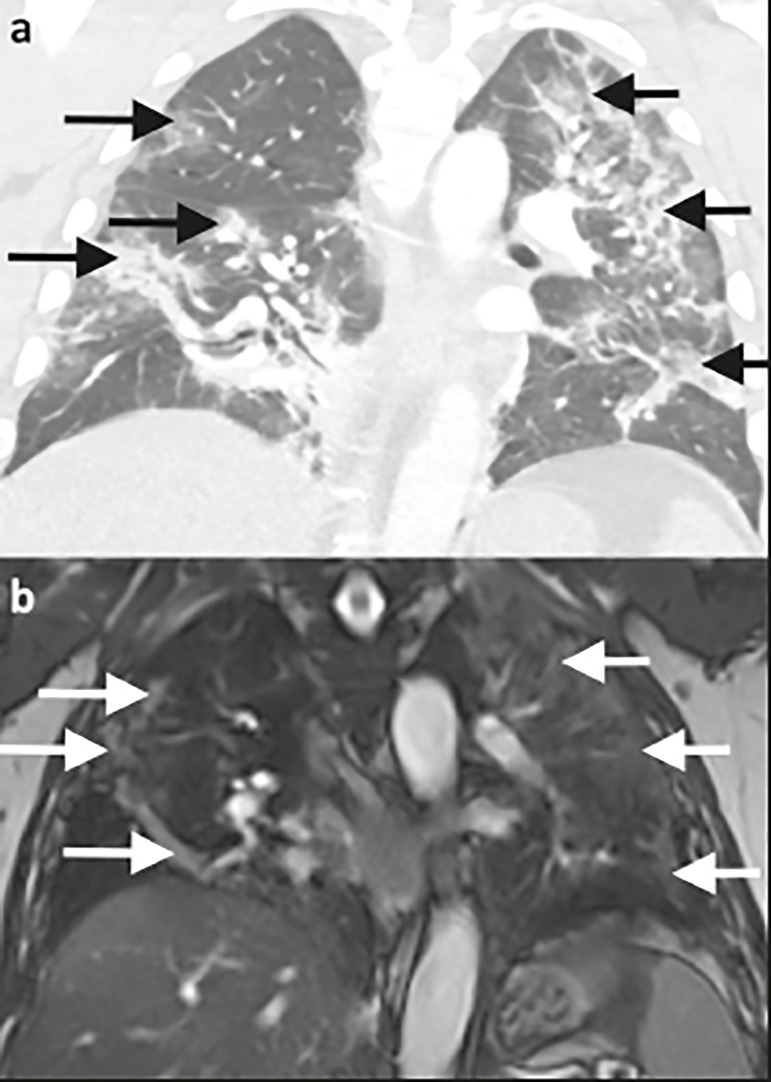
Ability of 1.5 T TrueFISP MRI to show the distribution of pulmonary findings. CT and MRI scans (a and b, respectively) showing patchy and linear consolidations and ground-glass opacities in the upper, middle, and lower lobes, as well as in the central and peripheral zones of the lungs (arrows). The CT scan was acquired on the day of COVID-19 confirmation by RT-PCR. MRI was performed 17 days after the CT.

**Figure 2:**
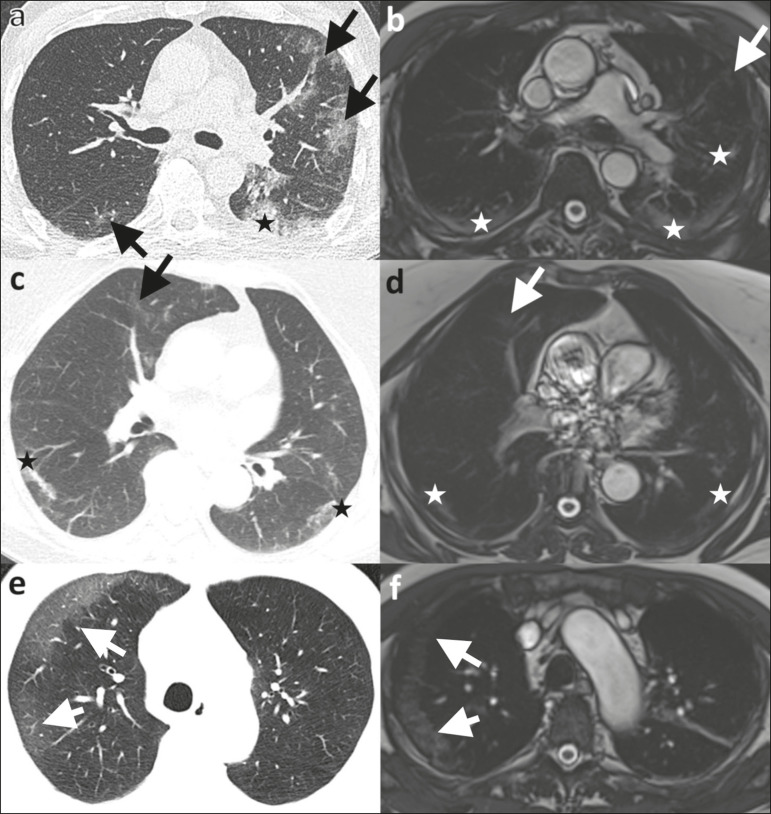
Ability of 1.5 T TrueFISP MRI to detect consolidations and ground-glass opacities. CT scan (a) showing peripheral ground-glass opacities of the left upper and right lower lobe (arrows), as well as consolidations of the left lower lobe (star), in a patient who was diagnosed with COVID-19 via RT-PCR two days later. MRI of the chest (b), performed 6 days after CT, showing consolidations in the lower lobes and left upper lobe (stars), with barely visible ground-glass opacities (arrow). Chest CT (c) of another patient, performed 14 days after confirmation of COVID-19 by RT-PCR, showing ground-glass opacities (arrow) and consolidations (stars), which are almost equally as visible on an MRI scan (d) acquired 2 days earlier. In another patient with COVID-19, ground-glass opacities with subpleural sparing in the right upper lobe (arrows) are present on a CT scan (e) performed on the day of COVID-19 confirmation by RT- PCR, as well as on an MRI scan (f) acquired 4 days later.

All 20 patients showed pulmonary abnormalities on CT scans, as well as on MRI scans. The distribution and appearance of the pulmonary findings on CT and MRI are presented in Table 2. The only significant difference between the two methods was that ground-glass opacities were visualized less often on MRI scans than on CT scans (*p* = 0.031). Bronchiectasis, bronchial wall thickening, and mucus plugging were seen on CT in 5%, 15%, and 15% of the patients, respectively, and on MRI in 5%, 5%, and 5%, respectively. Thoracic lymphadenopathy and pleural effusions were seen on 30% and 35% of the CT scans, respectively, compared with 30% and 40% of the MRI scans, respectively. The septal thickening and crazy-paving pattern seen on 20% of the CT scans was not detected on MRI scans, whereas linear/reticular pulmonary opacities were visualized almost as often on CT as on MRI (in 85% and 80% of the cases, respectively). Representative pulmonary findings and comparisons between CT and MRI findings are presented in Figures 1 and 2.

**Table 2:** Distribution and appearance of thoracic findings on CT and MRI scans of patients with COVID-19 pneumonia (N = 20).

	CT	MRI	
Parameter	n (%)	n (%)	*P*-value
Location			
Unilateral	1 (5)	2 (10)	1.000
Bilateral	19 (95)	18 (90)	1.000
Unilobar	1 (5)	2 (10)	1.000
Multilobar	19 (95)	18 (90)	1.000
Upper zone	16 (80)	17 (85)	1.000
Middle zone	19 (95)	19 (95)	1.000
Lower zone	20 (100)	19 (95)	1.000
Anterior zone	18 (90)	17 (85)	1.000
Posterior zone	20 (100)	20 (100)	1.000
Central zone	18 (90)	14 (70)	0.219
Peripheral zone	20 (100)	20 (100)	1.000
Subpleural sparing	10 (50)	5 (25)	0.180
Affected lobes			
Right upper	18 (90)	15 (75)	0.250
Middle	16 (80)	13 (65)	0.250
Right lower	19 (95)	20 (100)	1.000
Left upper	16 (80)	16 (80)	1.000
Left lower	19 (95)	18 (90)	1.000
Opacity			
Ground-glass	20 (100)	14 (70)	0.031
Consolidation	19 (95)	20 (100)	1.000
Inter-/intralobular septal thickening	4 (20)	0 (0)	0.125
Appearance			
Patchy/segmental	20 (100)	20 (100)	1.000
Reversed halo sign	3 (15)	4 (20)	1.000
Rounded	4 (20)	1 (5)	0.250
Linear/reticular opacities	17 (85)	16 (80)	1.000
Crazy-paving pattern	4 (20)	0 (0)	0.125
Air bronchogram	7 (35)	4 (20)	0.250
Bronchial changes			
Bronchiectasis	1 (5)	1 (5)	1.000
Bronchial wall thickening	3 (15)	1 (5)	0.500
Mucus plugging	3 (15)	1 (5)	0.500
Other			
Pleural effusion	7 (35)	8 (40)	1.000
Hilar/mediastinal lymphadenopathy	6 (30)	6 (30)	1.000

**Figure 3:**
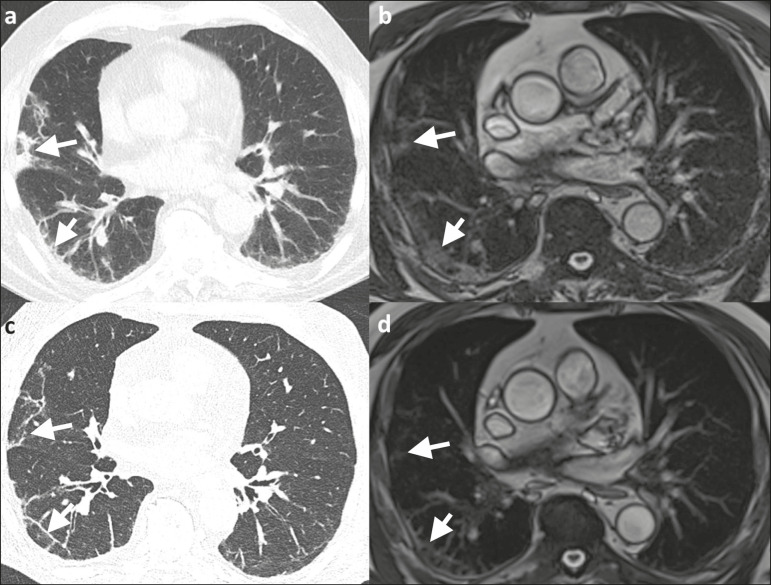
Appearance of pulmonary changes in COVID-19 pneumonia on 1.5 T TrueFISP MRI over time. Chest CT (a) of a patient who was diagnosed with COVID-19 via RT-PCR 28 days earlier, showing patchy and reticular consolidations in the middle lobe and right lower lobe (arrows). The opacities are almost equally as visible on an MRI scan (b) performed 8 days later. Follow-up CT scan (c) and follow-up MRI scan (d) acquired 56 and 59 days, respectively, after the RT-PCR almost equally show the reduction of the opacities to linear residuals (arrows).

As previously mentioned, eight patients underwent follow-up MRI, which showed that the pulmonary opacities had decreased in size, and quantity. Only one of those patients underwent follow-up CT, and the follow-up data were therefore not included in the statistical analysis. However, representative images are shown in Figure 3.

## DISCUSSION

The results of the present study indicate that it is possible to use TrueFISP MRI sequences to detect pneumonia caused by SARS-CoV-2 infection. On CT and MRI, the predominant findings were patchy/segmental and linear/reticular ground-glass opacities and consolidations, with a multilobar, bilateral distribution, in the peripheral zone of the lungs. The right lower lobe was most often affected. Regarding the distribution and appearance of the pulmonary findings, as well as bronchial changes, pleural effusion, and thoracic lymphadenopathy, no statistically significant difference was found between the MRI and CT scans. Although ground-glass opacities were visualized significantly less often on MRI scans than on CT scans, there were no statistically significant differences in terms of the visualization of consolidations and interlobular/intralobular septal thickening.

Our results are in line with those of other studies. The appearance and distribution of pulmonary abnormalities in our sample (i.e., the predominance of multilobar, peripheral ground-glass opacities and consolidations with a patchy and linear appearance) are typical manifestations of COVID-19 pneumonia described in the current literature^(7-9,23)^. The frequency of bronchial pathologies in our patient sample also coincides with that reported in other recent studies. Bernheim et al.^(7)^ reported bronchiectasis, bronchial wall thickening, and mucus plugging in 0.8%, 11.6%, and 0.8% of the patients evaluated, respectively, and Inui et al.^(23)^ found bronchial abnormalities in a total of 28%. In the present study, thoracic lymphadenopathy and pleural effusions were more common than has been reported in other studies on the subject^(7,9,23)^. However, the current knowledge on COVID-19 pneumonia suggests that such findings are indicative of greater severity^(2)^. All of the patients in our sample had suspected myocardial injury and therefore a rather severe disease course, which could explain the higher prevalence of thoracic lymphadenopathy and pleural effusion in our sample.

Our comparison of CT and MRI revealed broad agreement between the two modalities, except for the visualization of ground-glass opacities: We detected pulmonary abnormalities on all scans and found no statistically significant difference between CT and MRI concerning the distribution of pulmonary findings in patients with COVID-19 pneumonia. Accordingly, Attenberger et al., evaluating the suitability of 3-T MRI for the diagnosis of pneumonia in patients with neutropenia, reported that MRI and high-resolution CT are comparable in terms of overall lesion localization. Although MRI produced some false-positive and false-negative results in that study, those results did not alter the treatment strategy, because the pulmonary abnormalities were disseminated in all patients^(16)^.

In our sample, CT was slightly better at revealing septal thickening and the crazy-paving pattern than was MRI, although the differences were not statistically significant, whereas the two methods were comparable in terms of their ability to detect linear/reticular opacities. Linear/reticular opacities are usually more pronounced than are septal thickening and the crazy-paving pattern, and previous studies have shown that, when compared with CT, thoracic MRI has lower sensitivity for the detection of small pulmonary lesions^(5,9)^. In addition, linear/reticular opacities represent a stage of COVID-19 pneumonia later than that represented by septal thickening and the crazy-paving pattern^(11)^. Therefore, the discrepancy in the identification of linear/reticular opacities, septal thickening, and the crazy-paving pattern on MRI scans is presumably attributable to the lower sensitivity of TrueFISP MRI sequences for the identification of subtle pulmonary abnormalities and the time interval between the acquisition of CT and MRI scans in our study.

Regarding opacities, the authors of several other studies have also reported that the sensitivity of MRI is equivalent to that of CT for the detection of consolidations, although not for ground-glass opacities, some of which cannot be seen on MRI^(16,24)^. Recently, Singh et al.^(21)^ evaluated the diagnostic accuracy of MRI for respiratory infections in immunocompromised patients. Their MRI protocol, like ours, included a TrueFISP sequence, and the authors found it to have a sensitivity of 100% for the detection of consolidations and of only 16.6% for the detection of ground-glass opacities. Possible explanations for this phenomenon are the relatively low proton density of ground-glass opacities relative to that of consolidations, signal loss due to T2* decay, and cardiac motion. Promising results regarding the visualization of ground-glass opacities have been reported for low-field MRI, the low susceptibility of which results in higher signal intensity of lung parenchyma, as well as for respiratory-gated ultra-short echo time (UTE) MRI, which is less susceptible to T2* decay and motion artifacts than are other sequences^(22,25)^. Yang et al.^(14)^ examined the potential of UTE-MRI and CT for assessing COVID-19 pneumonia and reported that the two modalities provide similar image quality and high concordance for assessing the representative image findings. In particular, the lesion-based agreement between CT and UTE-MRI for evaluating ground-glass opacities was excellent (kappa: 0.815). For interlobular/intralobular septal thickening and the crazy-paving pattern, which were not visible on the MRI scans in our study, the level of intermethod agreement was moderate (kappa: 0.564) in the Yang et al.^(14)^ study. Heiss et al.^(18)^ reported a case in which persistent pulmonary abnormalities after COVID-19, including ground-glass opacities, were precisely visualized on low-field (0.55-T) MRI. The results reported by Heiss et al.^(18)^ and Yang et al.^(14)^ are promising with regard to using MRI for the diagnosis and surveillance of COVID-19 pneumonia in the future. However, the use of complex, nonstandard sequences and scanner systems for the optimal detection and characterization of subtle pulmonary abnormalities like ground-glass opacities was not the aim of our study. Rather, we wanted to determine whether COVID-19 pneumonia can be detected on standard MRI protocols, without the acquisition of dedicated lung sequences, carried out for reasons other than the investigation of pneumonia.

Although we were able to detect bronchial wall thickening and mucus plugging more often on CT than on MRI (in 15% and 5% of the cases, respectively), the difference did not reach statistical significance. In our sample, bronchiectasis was seen on one CT scan and one MRI scan. Arslan et al.^(26)^ reported comparable results for MRI in patients with primary immunodeficiency. The similar performance of CT and MRI for the detection of pleural effusion and thoracic lymphadenopathy, as observed in our study, has also been described in previous studies^(15,22,27)^.

Our study has some limitations. Among the patients included, the course of the disease was rather severe, because they all had suspected myocardial injury. That probably led to the extensive pulmonary abnormalities, which are not representative of all affected individuals. In addition, the relatively small number of patients limits the statistical power of the analyses. Nevertheless, our findings are in line with those of previous studies, which is why we assume that our results are reliable. Another limitation are the relatively long and varied time intervals between RT-PCR tests and imaging, as well as between CT and MRI examinations. Because the presentation, location, and extent of pulmonary abnormalities in COVID-19 pneumonia change, the comparability between CT and MRI findings is reduced over time^(7)^. For a lesion-based approach (i.e., a direct comparison of the appearance of single lesions, especially ground-glass opacities), a shorter time interval between the CT and MRI examinations would be essential. In this context, state-of-the-art sequences dedicated to lung imaging should be examined. The aim of our study, however, was to evaluate whether the pulmonary manifestations of COVID-19 pneumonia are generally visible and basically show the typical pattern described for CT on the widely used and available TrueFISP MRI sequences. As in larger studies, the CT and MRI scans in our study showed the typical appearance of COVID-19 pneumonia on CT, in all stages of the disease^(9)^, and the MRI findings in our study not only correlated well with those of other studies but also with the CT findings. We were therefore able to show that in the setting of the pandemic, pulmonary abnormalities due to infection with SARS-CoV-2 can be incidentally recognized as a COVID-19 pattern on MRI scans performed for other reasons.

## CONCLUSION

In conclusion, we were able to show that the pulmonary changes caused by COVID-19 pneumonia on TrueFISP MRI sequences are consistent with the patterns known from CT, and that such abnormalities can be detected on cardiac MRI. Given the special setting of the current pandemic, MRI examinations that show portions of the lungs should be carefully evaluated by the attending radiologists to promote the identification and isolation of unknown COVID-19 cases, thus curbing further spread of the disease. The results obtained encourage further detailed research to determine the value of lung MRI for the diagnosis of COVID-19 pneumonia.
